# Using artificial intelligence to learn optimal regimen plan for Alzheimer’s disease

**DOI:** 10.1093/jamia/ocad135

**Published:** 2023-07-18

**Authors:** Kritib Bhattarai, Sivaraman Rajaganapathy, Trisha Das, Yejin Kim, Yongbin Chen, The Alzheimer’s Disease Neuroimaging Initiative, The Australian Imaging Biomarkers and Lifestyle Flagship Study of Ageing, Qiying Dai, Xiaoyang Li, Xiaoqian Jiang, Nansu Zong

**Affiliations:** Luther College, Decorah, Iowa, USA; Mayo Clinic, Rochester, Minnesota, USA; University of Illinois Urbana-Champaign, Champaign, Illinois, USA; University of Texas Health Science Center, Houston, Texas, USA; Mayo Clinic, Rochester, Minnesota, USA; Mayo Clinic, Rochester, Minnesota, USA; Mayo Clinic, Rochester, Minnesota, USA; University of Texas Health Science Center, Houston, Texas, USA; Mayo Clinic, Rochester, Minnesota, USA

**Keywords:** Alzheimer’s disease, treatment learning, reinforcement learning, Q-learning, policy iteration, reward, action, policy

## Abstract

**Background:**

Alzheimer’s disease (AD) is a progressive neurological disorder with no specific curative medications. Sophisticated clinical skills are crucial to optimize treatment regimens given the multiple coexisting comorbidities in the patient population.

**Objective:**

Here, we propose a study to leverage reinforcement learning (RL) to learn the clinicians’ decisions for AD patients based on the longitude data from electronic health records.

**Methods:**

In this study, we selected 1736 patients from the Alzheimer’s Disease Neuroimaging Initiative (ADNI) database. We focused on the two most frequent concomitant diseases—depression, and hypertension, thus creating 5 data cohorts (ie, Whole Data, AD, AD-Hypertension, AD-Depression, and AD-Depression-Hypertension). We modeled the treatment learning into an RL problem by defining states, actions, and rewards. We built a regression model and decision tree to generate multiple states, used six combinations of medications (ie, cholinesterase inhibitors, memantine, memantine-cholinesterase inhibitors, hypertension drugs, supplements, or no drugs) as actions, and Mini-Mental State Exam (MMSE) scores as rewards.

**Results:**

Given the proper dataset, the RL model can generate an optimal policy (regimen plan) that outperforms the clinician’s treatment regimen. Optimal policies (ie, policy iteration and Q-learning) had lower rewards than the clinician’s policy (mean −3.03 and −2.93 vs. −2.93, respectively) for smaller datasets but had higher rewards for larger datasets (mean −4.68 and −2.82 vs. −4.57, respectively).

**Conclusions:**

Our results highlight the potential of using RL to generate the optimal treatment based on the patients’ longitude records. Our work can lead the path towards developing RL-based decision support systems that could help manage AD with comorbidities.

## INTRODUCTION

Alzheimer’s disease (AD) is a progressive neurological disorder causing cognitive impairment and brain atrophy. Approximately 5.8 million people in the United States age 65 years and older live with AD and approximately 60%–70% of 50 million people worldwide with dementia are estimated to be diagnosed with AD.^1^ Currently, the exact etiology of AD is still unknown.^2^ β-Amyloid plaque formation and aggregation,^2^ apolipoprotein E (Apo E) gene along with various environmental factors^3^ could be involved in the AD pathogenesis and additional risk factors; like vascular diseases, type-2 diabetes, traumatic brain injury, epilepsy, depression, smoking, diet, physical exercise, and alcohol consumption^4^ could be involved in the dementia pathogenesis. Due to the unknowns of AD’s etiology and risk factors, drug development has not made any significant progress and available drugs like cholinesterase inhibitors (ChEIs) and memantine only treat the disease superficially. These drugs only help to temporarily ameliorate memory and thinking problems, but they do not treat the root cause of AD nor slow the rate of decline of a patient’s condition.^5^ They are aimed at modifying just the disease symptoms.^6^^,^^7^

AD management is further complicated by the high rate of comorbidities observed in patients.^8^ Approximately 90% of AD patients are diagnosed with comorbid conditions,^9^ and the large majority with chronic diseases such as hypertension and depression.^10^^,^^11^ Patients are very often treated with medications for other comorbidities. The relationship between AD and these comorbid conditions warrants further investigation on whether they act as risk factors or by-products of AD, which further complicates the management of AD. Medication management ends up being a trial until a regimen temporarily relieves symptoms. As a result, it could take years of experience for a physician to medically manage AD with comorbidities.^12^ Instead of trialing different regimens for temporary symptom relief, a medication regimen learning tool can be beneficial in providing junior physicians with the necessary information to best treat AD patients diagnosed with comorbidities. The tool can suggest individualized drug combinations based on patients’ state, rather than having physician’s trial several medications. This would increase time efficiency in selecting the best treatment option; thus, equipping physicians with the resources to provide the best, timely care for patients.

Various artificial intelligence (AI) techniques have been used to create tools for detecting AD.^13^^,^^14^ The authors in Ref.^15^ report a multimodal recurrent neural network to predict conversions from mild cognitive impairment (MCI) to AD using longitudinal biomarkers as well as cross-sectional neuroimaging data. The use of efficient convolutional neural network architectures using a small number of parameters to prevent overfitting yielded high MCI to AD predictive performance (average AUC of 0.925) in Ref.^16^ To improve early-stage AD diagnosis, the authors in Ref.^17^ provide a data augmentation strategy to reduce overfitting problems. Further, their model generates a heatmap on brain images to improve explainability. Reinforcement learning (RL) has been used to predict and model 10-year cognition trajectories.^18^ While multiple studies exist for the diagnosis, subtyping, drug repurposing, and biomarker identification of AD,^19^ there is a dearth of studies involving AI tools for optimizing treatment regimens for AD patients.

**Figure 1.**
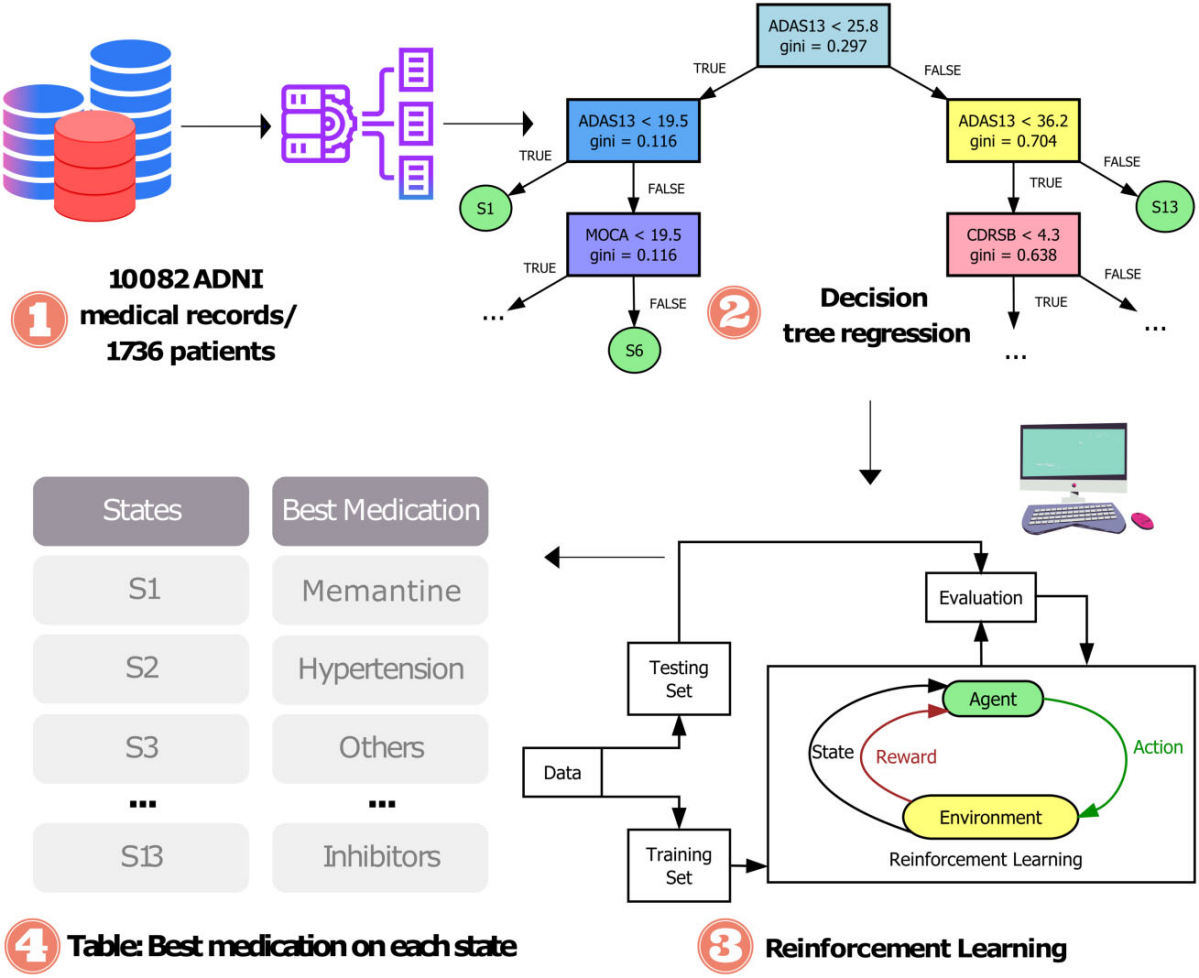
Pipeline of reinforcement learning-based regimen plan. (1) The raw data that store all the scores of tests like Alzheimer’s Disease Assessment Scale (ADAS), Montreal Cognitive Assessment (MoCA), Clinical Dementia Rating Scale Sum of Boxes (CDRSB), age, and so on. It also stores the medication applied and rewards based on Mini-Mental State Exam (MMSE) score. (2) Thirteen different states are defined using the decision tree. (3) A reinforcement learning model is prepared based on states from subfigure (2) and actions and rewards from data in subfigure (1). (4) Best medication/action is selected for each state after using reinforcement learning.

AI has made it possible to create medication regimen learning tools. Recently, it has been used to create such decision-support system models to predict drugs based on patient reviews.^20^ RL is an AI technology to learn a set of actions that can reward the most during the interaction of an agent in a specific environment (eg, a computer game). RL has achieved great success in diverse applications that require human interactions (eg, Go^21^), suggesting its capability of learning human-ish behavior. Healthcare is quickly adapting RL into their systems, as seen in regimen plans learned from Parkinson’s disease (PD)^21^ and Sepsis.^22^ This technology can learn from existing clinical data to provide senior-level experience to junior physicians with less experience, potentially revolutionizing the transfer of information in healthcare. To this end, we propose a study to learn a RL-based model for the clinical practice of junior clinicians in managing AD patients. This model consists of states, actions, and rewards, and is designed to check the current state, explore different actions, and pick the one that maximizes future rewards (Figure 1). This model outperforms traditional data-derived methods, such as the transition probability-based model, particularly for patients with concomitant conditions (ie, depression and hypertension). This is evidenced by the comparison of the Mini-Mental State Exam (MMSE) scores from the data to the MMSE predicted by our RL model. The results of our study demonstrate that the proposed model can generate a clinician’s regimen plan for AD patients.

This work makes several valuable contributions to the field. Firstly, it breaks new ground by applying RL to learn treatment regimens for AD at the level of clinicians. This innovative approach opens up exciting possibilities for optimizing patient care and treatment outcomes. Secondly, the development of models that generate treatment plans for AD, Hypertension, and Depression represents a significant advancement in addressing the complex nature of multiple comorbidities in patients. By considering these interconnected conditions, the models offer a more comprehensive approach to personalized healthcare. Thirdly, the extensive testing of these models across 2 large longitudinal datasets, namely the Alzheimer’s Disease Neuroimaging Initiative (ADNI) and the Australian Imaging, Biomarkers & Lifestyle Flagship Study of Ageing (AIBL) databases, ensures robustness and generalizability of the findings. This thorough evaluation provides a solid foundation for further research and clinical implementation. Lastly, the provision of an open-source Python-based toolbox not only promotes transparency and reproducibility but also empowers the scientific community to build upon and enhance the existing work. Overall, these contributions collectively advance our understanding and potential treatment options for AD and its associated comorbidities.

## MATERIALS AND METHODS

### Data

The data are derived from ADNI database (adni.loni.usc.edu), the most frequently used open-access data in the pharmacogenomic studies for AD.^14^ ADNI is a longitudinal multicenter study designed to support advances in AD prevention and treatment by developing clinical, imaging, genetic, and biochemical biomarkers.^23^ Data used in the preparation of this article were obtained from the ADNI database (adni.loni.usc.edu). The ADNI was launched in 2003 as a public-private partnership, led by Principal Investigator Michael W. Weiner, MD. The primary goal of ADNI has been to test whether serial magnetic resonance imaging, positron emission tomography, other biological markers, and clinical and neuropsychological assessment can be combined to measure the progression of MCI and early AD. For up-to-date information, see www.adni-info.org. For testing the generalizability of our models, we used the AIBL database (https://aibl.csiro.au/). Data were collected by the AIBL study group. AIBL study methodology has been reported previously (Ellis et al. 2009).^24^

### RL-based modeling

The traditional medical method of treating AD is assessing a patient’s current state and prescribing medication accordingly, then following up on symptoms afterward. We utilized RL to measure AD progression based on selected consecutive decisions. This consecutive decision-making nature of RL models is best described as a Markov decision process. A Markov decision process consists of states, actions, and rewards where a state is Markovian if and only if the next state is dependent only on the current state. It is based on an agent at a certain state selecting different actions to maximize the rewards. The defined factors are described below.

#### State s

We define states as a finite set of a patient’s progression state in the latest clinic visit. Raw data on participants’ states were converted to discrete states. We picked up statistically significant features like Alzheimer’s Disease Assessment Scale (ADAS13) and age (Table 1) to predict the MMSE score using regression. We then chose the significant variables and derived a decision tree (Supplementary Figure S5 and Table S1) to predict the MMSE scores. The decision tree divides each data into different ranges and then predicts the MMSE score. For example, an age of fewer than 70 years and an ADAS score of more than 20 could predict an MMSE score of 20. The predicted MMSE scores at the leaf nodes of a decision tree are our derived discrete states (Supplementary Figure S5). We grouped each visit according to the criteria specified by the decision tree and ignored states with less than 50 occurrences to avoid states without enough visits.

**Table 1.** Disease states classification based on a decision tree

Data cohort	Disease state							
	State	ADAS13	RAVLT immediate	RAVLT learning	Age	CDRSB	MOCA	FDG
Whole Data	S0	(,19.5]	(,22.5]		(,77]			
	S1	(,19.5]	(,22.5]		[78,)			
	S2	(,19.5]	(22.5,)			(,1.8]		
	S3	(,19.5]	(22.5,)			(1.8, ]		
	S4	(19.5, 25.8]				(,1.8]	(,19.5]	
	S5	(19.5, 25.8]				(1.8, ]	(,19.5]	
	S6	(19.5, 25.8]					(19.5, 21.5]	
	S7	(19.5, 25.8]					(21.5, ]	
	S8	(25.8, 36.2]				(, 1.8]	(,19.5]	
	S9	(25.8, 36.2]				(1.8, 4.2]	(,19.5]	
	S10	(25.8, 36.2]				(4.2, ]	(,19.5]	
	S11	(36.2, 42.7]						
	S12	(42.7,)						
AD	S13	(,18.5]		(,1.5]				
	S14	(,18.5]		(1.5,)				
	S15	(18.5, 25.2]					(,21.5]	(,6]
	S16	(18.5, 25.2]					(,21.5]	(6,)
	S17	(18.5, 25.2]					(21.5,)	
	S18	(25.2, 35.8)				(,3.2]		
	S19	(25.2, 35.8)				(3.2,)		(,5]
	S20	(25.2, 35.8)				(3.2,)		(5,)
	S21	(35.8,)						
AD-Hypertension/ AD-Hypertension-Depression	S22	(,17.5]					(,20.5]	
	S23	(17.5, 22.2]					(,20.5]	
	S24	(, 22.2]					(20.5,)	
	S25	(22.2, 25.2]					(, 13.5]	
	S26	(22.2, 25.2]					(13.5,)	
	S27	(25.2, 31.2]				(,1.8]		
	S28	(25.2, 31.2]				(1.8, 3.2]		
	S29	(25.2, 31.2]				(3.2,)		
	S30	(31.2, 41.8]			(,75]			
	S31	(31.2, 41.8]			(75,)			
	S32	(41.8,)						
	S33	(25.2, 31.2]				(,3.2]		
AD-Depression	S34	(, 22.2]				(, 3.75]	(, 20.5]	
	S35	(, 22.2]				(3.75,)	(, 20.5]	
	S36	(, 22.2]					(20.5,)	
	S37	(22.2, 25.2]				(,3.75]	(, 21.5]	
	S38	(22.2, 25.2]				(3.75,)	(, 21.5]	
	S39	(25.2, 35.8]				(,3.25]		
	S40	(25.2, 35.8]				(3.25,)		
	S41	(35.8,)					(,12.5]	
	S41	(35.8,)					(12.5,)	

#### Action a

We defined actions as a finite set of medications. Six combinations of drugs based on usage frequency were used: ChEIs, memantine, ChEIs+memantine, antihypertensive drugs, other supplements, and no drugs. Hypertension drugs and other supplements are also included to explore treatment across 5 data cohorts: Whole, AD, AD-Hypertension, AD-Depression, and AD-Depression-Hypertension. Please note that hypertension drugs and supplements are not traditional treatments for AD and are for patients with coexisting hypertension and other conditions.^25^^,^^26^

#### Reward r

We defined reward as the clinical assessment of the patient’s medication response. While multiple assessment scores are used in clinical practice (eg, Rey Auditory Verbal Learning Test [RAVLT] tests, Montreal Cognitive Assessment [MoCA]), we used MMSE assessment scores in our study because it is a widely used tool to assess cognitive function in both routine clinical practice and research settings.^27^^,^^28^ The max score for MMSE is 30 points, with ranges from 20 to 24 indicating mild dementia; 13 to 20 indicating moderate dementia, and less than 12 indicating severe dementia.^29^ We calculated the difference between MMSE in the current visit and the previous visit to measure the rate of progression of AD. A discount rate gamma, 0≤ γ ≤1 was also introduced to determine the present value of future rewards.^30^ We used the discount factor γ = 0.3. Our total discounted return is represented by:


$$G_{t}=R_{t+1}+\gamma \;R_{t+2}+\gamma^{2}\;R_{t+3}+\cdots =\sum_{k=0}^{\infty }\gamma^{k}*\;R_{t+1+k}.$$

### Policies

The policy is a map from state to action. It maps an action to every possible state in the system. In other words, it can be described as a possible strategy an agent uses in each state to get rewards and it is defined by probability. For example, if an agent uses an action a_1_ on state s_1_ and a_2_ on state s_2,_ and so on, it can be considered a policy of the agent. On the state action map, for state s_1_, a_1_ has the highest probability value and for state s_2,_ a_2_ has the highest probability value. There are many possible policies as different actions can be used for the same states; however, one policy will yield the maximum reward.

#### Optimal policy learned by RL learning

We generated policies using 2 different RL methods—model-free Q-learning and model-based policy iteration. Model-free Q-learning is an algorithm that uses trial and error to learn the best action to take in a given state. It does not require any prior knowledge of the environment and can be used to solve complex problems. Model-based policy iteration, on the other hand, uses a model of the environment to determine the best action to take. It requires prior knowledge of the environment and can be used to solve problems more efficiently. Here, the prior knowledge of the environment is encoded by the transition state probabilities estimated from the training data. Model-free Q-learning is more general and can be used in a variety of situations, while model-based policy iteration is more specific and can be used to solve problems more quickly. Model-based methods rely on planning and transition probabilities, while model-free methods rely on learning or experience.^30^

##### Policy iteration

First, we compute the state-value function v(s) for an arbitrary policy **π**. Value function, v(s) is a function that estimates future rewards on a given state when performing a particular action based on transition probability. The transition probability is the probability of transitioning from one state, s, to another state, s′ after a certain action is applied. This is called policy evaluation. After computing the value function for a policy, we check if there is a particular action that gives a better value for that state. This is repeated until a better policy is found and is called policy improvement. We repeat these evaluation and improvement cycles until we find out the optimum policy.

##### Q-learning

We used the off-policy temporal difference algorithm to create more variety for optimal policies. Q-learning uses Q-value from a Q-table to find the best actions for each state. The Q-value is an estimation of how good an action is at a particular state. The Q-table is an m*n matrix where m is the number of states and n is the number of actions. An agent applies an action at a particular state and updates the Q-table with the reward it receives for that state-action combination. Then the agent applies different actions for the same state. Through numerous repetitions, the best action for each state is picked and the Q-table becomes stable. The speed at which Q-table is updated is dependent on a parameter alpha, 0≤α<, 1 the learning rate. We set our alpha to 0.05 so that the Q-table converges after enough trials. It is different from policy iteration because it gives an optimal policy independent of the policy being followed. In other words, it is not dependent on transition probability derived from the dataset.

#### Clinicians’ policy by a data-driven approach

We used transition probability to find the clinician’s policy from the data. We followed an approach similar to policy iteration. We used policy evaluation and policy improvement process just once based on the existing transition probability from the data and made the resultant policy as the clinician’s policy. Since the policy is totally based on the data, we can safely assume it is very close to the real clinician’s policy.

#### Other policies

We also created zero policy and random policy to compare them with our RL-based and clinician policies. Zero policy implies that in each state no drugs are applied as actions and random policy implies that random drugs are applied as actions without assessing the patient’s condition.

### Experiment design and evaluation

#### Evaluation and comparison

We used offline evaluation to estimate the value of target policies (policies being learned) based on a behavior policy (policy used to generate behavior) derived from the offline log data. It is very useful in settings where online interaction involves high risks and costs (eg, medication recommendation systems).^31^ We used importance sampling (IS), commonly used off-policy evaluations, to estimate expected values under one distribution given samples from another.^30^ It estimates the value of a target policy from behavior policy derived from the data by reweighing states based on the frequency of their occurrence.^32^ In our study, we used stepwise weighted importance sampling (step-WIS) which is the most practical point estimator among the importance of sampling techniques because of its low variance^21^^,^^33^ and error.^34^

#### Tests

- Test 1: The first test evaluated the impact of data size in generating policies from AD data in order to create a policy with a higher rate of accuracy and closest to the clinician’s policy. We split 60%/20%/20% for training, validation, and testing. With the training set, we further divided it into 4 scenarios relating to different data sizes (eg, 100%, 80%, 50%, 30%) to feed the models. All training groups were trained 50 times to generate an optimal policy. We repeated this cycle 100 times to eliminate any potential bias in our final reward. A total of 13 states and 6 actions were used for this test.
- Test 2: The second test evaluated how the proposed work will perform over the different patient cohorts (eg, patients with different concomitant diseases). We separated the data into 5 groups based on the disease diagnosis: AD (9 states, 6 actions), AD-Hypertension (10 states, 6 actions), AD-Depression (9 states, 6 actions), and AD-Depression-Hypertension (10 states, 6 actions). Hypertension and depression were the 2 most prevalent concomitant diseases among patients in the data. Also, depression is one of the most prevalent psychiatric conditions in AD patients.^35–37^ We then followed the same splitting method of 60%/20%/20% for training, validation, and testing, respectively. We also wanted to check how different RL’s medicine prediction is for different states compared to the clinician’s prediction.
  We tried to check the generalizability of our model by testing our algorithms on a different dataset. For this, we picked the AIBL dataset. In order for us to perform the model on this dataset, we had to find variables common to both the ADNI and AIBL datasets. We found this included Clinical Dementia Rating, Neuropsychological test scores, and laboratory screening data. Specifically, these were the common variables we used: “RID,” “VISCODE,” “AXT117,” “BAT126,” “HMT3,” “HMT7,” “HMT13,” “HMT40,” “HMT100,” “HMT102,” “CDGLOBAL,” “LIMMTOTAL,” “LDELTOTAL.” We trained our model on ADNI data with these variables and then tested them on the AIBL dataset. Our research indicated that the medication (actions) for AIBL data were not similar to the actions in ADNI data. Therefore, the AIBL was used for testing alone. We assigned actions to the AIBL dataset using the clinician policy obtained from the ADNI data. The data cohorting for AIBL were similar to the ADNI dataset. The results of these experiments are shown in Supplementary Figure S1.
- Test 3: For our third test, we wanted to see how our proposed Q-learning model performed with 2 different MMSE score (reward) cohorts, MMSE greater than and MMSE smaller than the average MMSE of all patients. First, we calculated the average MMSE of each patient for all their visits and then calculated the average MMSE of all the patients. If a patient’s average MMSE score for all the visits was less than the average of all patients, the patient was categorized as JR clinician patients and the remaining patients were categorized as SR clinician patients. We then compared the JR clinician-patient cohort and SR clinician-patient cohort with the whole patient cohort. We then followed the same splitting method used in Test 2.
- Test 4: For our fourth test, we wanted to learn how our proposed Q-learning model performed over different learning rates, α. This test was to confirm our Q-learning was robust enough to learn the real clinician’s policy. We used our already existing AD cohort and compared the results for alphas from 0.1 to 0.9. We then followed the same splitting method used in Test 2.
- Test 5: For our final test, we wanted to learn how our proposed Q-learning model performed over the different number of states while keeping the data constant. We changed the total number of discrete states given by a decision tree based on the number of samples. For example, for Whole Data, we got 13 states when we used leaf nodes of a decision tree that had more than 50 samples and 9 states with leaf nodes that had more than 200 samples and compared the results (Figure 6). We then followed the same splitting method used in Test 2.

## RESULTS

### Patient cohort

We selected patients based on the following criteria: a minimum of 2 clinic visits, complete medical history, and clinical assessment data (Table 2). A total of 1736 patients were selected (957 males and 779 females). Across all selected patients, the total number of visits was 10 082. The mean monthly visits and mean number of visits per patient were 32.17 months and 6.42 visits, respectively. The patient cohorts we selected are defined as follows:

**Table 2.** Patient demographics for the different cohorts of data (Whole Data, AD data, AD-Hypertension data, AD-Depression data, and AD-Hypertension-Depression data)

Features	Cohorts				
	Whole Data	AD	AD-Hypertension	AD-Depression	AD-Hypertension-Depression
No. of patients	1736	430	510	479	549
No. of visits (SD)	6.42 (3.64)	7.10 (3.65)	7.35 (3.81)	7.14 (3.66)	7.35 (3.18)
No. of months of follow-up (SD)	32.17 (25.75)	41.00 (26.89)	43.22 (27.79)	41.54 (26.87)	43.46 (27.74)
No. of male patients	957	257	305	283	324
No. of female patients	779	173	205	196	225

	**No. of Patients in Cohorts**				
**Medications**	**Whole data**	**AD**	**AD-Hypertension**	**AD-Depression**	**AD-Hypertension-Depression**

Supplements/others	4573	1176	1502	1415	1701
No drugs taken	4500	1016	1246	1124	1337
Cholinesterase inhibitors	418	418	418	418	418
Memantine	176	176	176	176	176
Antihypertensive drugs	270	125	270	147	270
Memantine-cholinesterase inhibitors	145	145	145	145	145

- The “Whole Data” cohort includes all patients, regardless of whether they have been diagnosed with any condition or prescribed any medication.
- The “AD” cohort comprises patients diagnosed with AD or those who have been prescribed medications specifically for AD. These patients may or may not have other medical conditions. It is important to note that we also include patients with MCI in the AD cohort.
- The “AD-Hypertension” cohort consists of patients diagnosed with or treated for AD (including MCI), as well as patients with hypertension who may have AD, MCI, or are cognitively normal (CN).
- The “AD-Depression” cohort includes patients diagnosed with or treated for AD (including MCI), as well as patients with depression who may have AD, MCI, or CN.
- The “AD-Depression-Hypertension” cohort includes patients diagnosed with or treated for AD (including MCI), patients with depression who may have AD, MCI, CN, as well as patients with hypertension who may have AD, MCI, or CN.

It is important to note that we have included CN patients in the AD-Hypertension, AD-Depression, and AD-Depression-Hypertension cohorts. This is because AD pathology can be present in individuals without evident memory loss, and these CN individuals may already exhibit subtle brain atrophy. Including these patients helps reduce bias and allows for training a more robust model.

### Test 1

**Figure 2.**
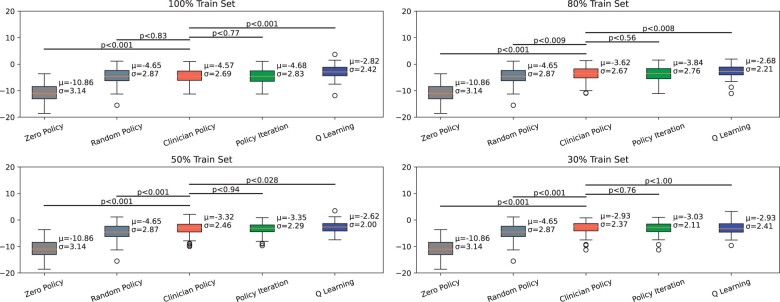
Comparison of rewards represented by MMSE score (*y*-axis) for different-sized data for all policies. Policy iteration and Q-learning are the optimal policies, and the clinician policy is derived from the data. The edge of the boxes represents the boundaries of the middle 2 quartiles of the data, the orange line represents the median, and the whiskers show the range of the data excluding the outliers. The Student’s *t* test is used to provide *P*-values between the different data groups shown.

Test one (Figure 2) revealed that appropriate data size resulted in RL performance comparable to the clinician’s performance. As the data samples increased, results in policy iteration and Q-learning displayed increasingly better results that are comparable, if not better than the clinician’s performance. For example, the 30% train set had a lower policy iteration score [mean = −3.03] and Q-learning score [mean = −2.93] than the clinician’s policy score [mean = −2.93]. This is in contrast to the performances with 100% train set, where the policy iteration [mean = −4.68] is at the level of and the Q-learning [mean = −2.82] outperforms the clinician’s policy [mean = −4.57]. A more detailed analysis (Supplementary Figure S7) reveals a cutoff of 50% training data, beyond which the Q-learning method outperforms the clinician policy. This increase in performance of the policy iteration algorithm, as well as the Q-learning algorithm demonstrates the scope for improvement with additional data. Overall, optimal policy consistently outperformed zero policy and random policy. Random policy [mean = −4.65] consistently outperformed zero policy which repeatedly yielded the lowest mean reward of −10.86.

The suggestions made by both optimal policies and clinicians’ policies are somewhat similar (Table 3). Both policy iteration and Q-learning start off by recommending no drugs when patients are in the first state whereas the clinicians recommend memantine. In State 11, all the policies recommend hypertension whereas, in State 12, the recommendation by each policy is completely different. In state 6, both optimal policies recommend hypertension whereas clinicians recommend memantine. Supplementary Figure S2 has different actions recommendation for each state for AD-Depression-Hypertension.

**Table 3.** Comparison of recommended action for policy iteration, Q-learning, and clinician’s policy for whole data

	Actions																	
	Clinicians						Q-learning						Policy iteration					
States	No	In	Me	Hy	Ni	So	No	In	Me	Hy	Ni	So	No	In	Me	Hy	Ni	So
S 0																		
S 1																		
S 2																		
S 3																		
S 4																		
S 5																		
S 6																		
S 7																		
S 8																		
S 9																		
S 10																		
S 11																		
S 12																		

*Note*: The actions are represented by different colors.

No: no drugs; In: inhibitors; Me: memantine; Hy: hypertension drugs; Ni: combination of memantine and inhibitors; So: supplements/other drugs.

### Test 2

**Figure 3.**
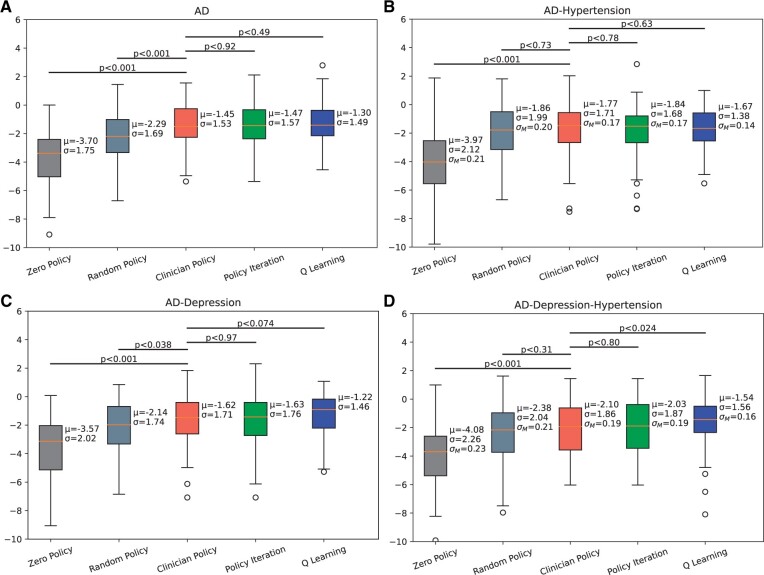
Comparison of different optimal policies (policy iteration and Q-learning) and the clinician’s policy for different concomitant disease cohorts. (A) Comparison for AD patients, (B) Comparison for AD patients with concomitant disease hypertension only, (C) Comparison for AD patients with depression, and (D) Comparison for AD patients with hypertension and depression.

We noticed that the model is comparable with the clinician’s policy when data are split around AD itself. Since hypertension and depression are frequently seen in AD patients and our actions are mainly the medication for AD, policy iteration outperformed the clinician’s policy in all 3 cohorts (Figure 3). We also concluded that Q-learning’s rewards are more coherent than clinicians’. For all the data cohorts, Q-learning’s reward predictions are scattered around 0 (lower negative values) whereas clinician reward predictions are scattered around higher negative values rewards (Supplementary Figure S6).

### Test 3

**Figure 4.**
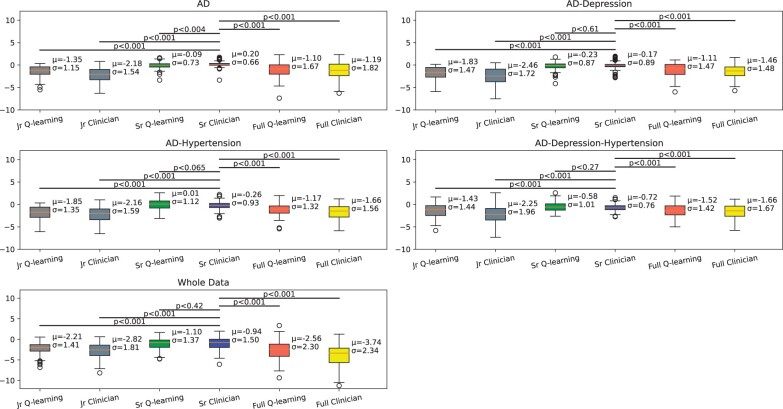
Comparison of clinician policy reward and Q-learning policy reward between JR clinician, senior clinician, and combined data cohorts.

We found that the SR clinician cohort outperformed the JR clinician cohort for all data cohorts. The difference between the SR clinician policy and the SR Q-learning policy was not consistent throughout the data cohort. In the AD data, the SR Q-learning policy [mean −0.09] had worse results than the SR Clinician policy [mean 0.20]. In the AD-Depression-Hypertension data cohort, the SR Q-learning policy [mean −0.58] outperformed the JR clinician policy [mean −0.72]. On the contrary, the JR Q-learning policy outperformed the JR Clinician policy across all data cohorts (Figure 4).

### Test 4

**Figure 5.**
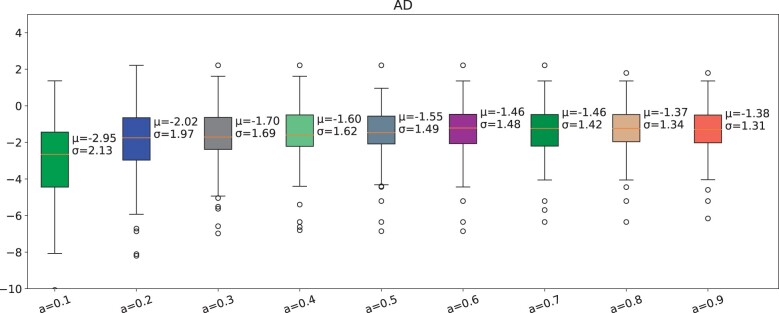
Comparison of Q-learning policy for different learning rates for AD cohort. The learning rate is from 0.1 to 0.9.

We also confirmed that the Q-learning policy is not always better with high learning rate (alpha) values. There is a general trend of increasing rewards from a learning rate of 0.1–0.4. Then, the reward is stable from the alpha value of 0.3 to around 0.8 with a mean from −1.28 to −1.30 and then it decreases at 0.9 with a reward of −1.42 (Figure 5).

### Test 5

**Figure 6.**
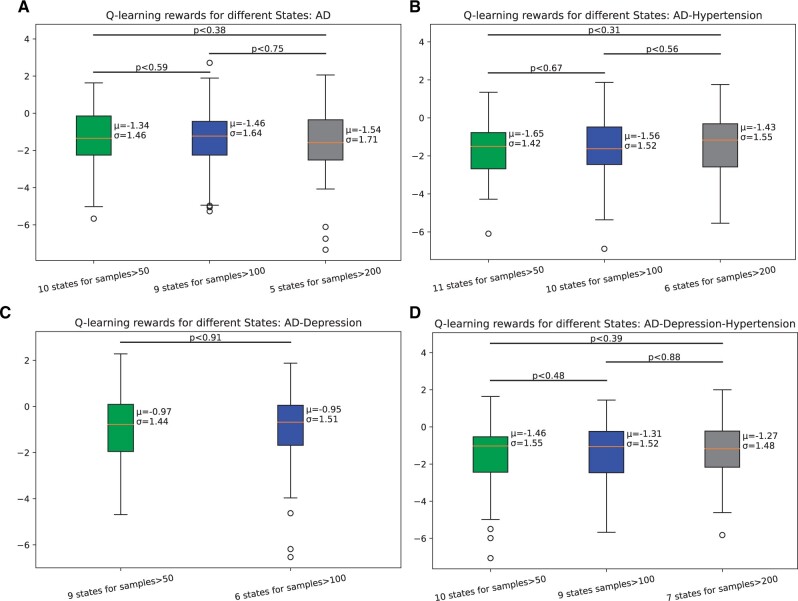
Comparison of Q-learning policy for the different number of states for different data cohorts. The number of states is based on the number of samples on the leaf node of a decision tree.

We did not find any concrete connection between changing the number of states and mean reward prediction (Figure 6). In the AD data cohort, 10 states from leaf nodes with a sample size greater than 50 predicted better mean reward [mean=−1.34] compared to 7 states from leaf nodes with a sample size greater than 200 [mean=−1.54] and 9 states from leaf nodes with a sample size greater than 100 [mean=−1.46]. In the AD-Depression data cohort, 9 states from leaf nodes with a sample size greater than 50 predicted worse mean reward [mean=−0.97] compared to 6 states from leaf nodes with a sample size greater than 100 [mean=−0.95] (Figure 6). This analysis for Whole Data cohort is in Supplementary Figure S4.

## DISCUSSION

Our current study proposed an RL-based model to investigate the optimal AD treatment regimen plan based on the electronic health record. We adopted 2 RL methods—model-free Q-learning and model-based policy iteration—to generate the regimen plans. In comparison to the policy (ie, treatment regimen plan) learned simply from the existing data (ie, clinician’s policy based on transition probability-based method), the experiments displayed RL models that can optimize the treatment regimen for AD given sufficient patient data as suggested by previous studies with Parkinson’s^21^ and sepsis.^22^ However, our current study has notable differences compared to those studies. First, we argue that the AI models can only estimate an optimal policy, which is not comparable to, nor substitutes for a real clinician’s policy. This is unlike previous studies that strongly suggest AI-based policies can outperform physician policies.^21^ Secondly, in previous studies, all the policies were generated based on the on-policy methods (eg, SARSA and value interaction^21^^,^^22^), which consider the target policy to be identical to the behavior policy. This is problematic in an offline setting because our target policy is very different from the behavior policy as we are using different actions for different states in order to find an optimal action for a particular state. As a response, we conducted an evaluation that fairly compared the offline model-free models (ie, Q-learning) with the behavior policy. Lastly, we incorporated the importance of data volume to learn an optimal model for real-world implementation in addition to focusing on the RL model performance. Experiments on different data cohorts revealed better RL-based model performance in larger data cohorts. Our experiment showed a harmonization should be achieved between the data and method to generate an optimal policy. In our study, we found the optimal policy by repeating experiments with the training and validation data 50 times. For generalizability, we used 100 bootstrap samples of training and testing data on the resulting optimal policy to find our final reward. This study provided a robust guide for treatment plan learning and has adaptable potential in guiding the treatment of AD patients for junior physicians.

Our results were promising and demonstrated high potential for RL-based models to learn real clinician’s policies; however, there are a few limitations to address. First, we could not obtain definitive results from the latest offline RL algorithm, like Conservative Q-Learning (CQL), as it consistently predicted supplements as the optimal action. This is due to the discrepancy in prescription frequency between supplements (N = 4573) and specific medications such as ChEIs (N = 418), memantine (N = 176), antihypertensive drugs (N = 270), and memantine-ChEIs (N = 145). The numbers reported here represent instances of prescriptions given during individual visits. Since patients are prescribed multiple drugs or supplements during the same visit or during multiple visits, there is no clear pathway to rebalance the data with respect to medications either via undersampling or via oversampling. For example, removing patient data who were prescribed supplements would also remove medication samples and vice versa. This is in contrast with previous studies examining PD which did not have higher rates of prescribed supplements (N = 442) compared to PD medications (Levodopa = 1157 and Dopamine agonist = 447). There is a lot of potentials to perform this study by using the latest RL algorithms like CQL if evenly distributed medication data are collected in the future.

A second limitation lies in the accuracy of calculating disease progression with only cognitive assessment data. We could not incorporate neuroimaging and other biomarkers data as these were not available. Although there is no precise way to measure the progression of AD, neuroimaging has been widely used to diagnose AD and monitor disease progression.^38^ Due to the unavailability of such data, we had to rely on commonly used cognitive tests like MMSE, ADAS, and CDRSB. A more in-depth study can be performed by incorporating other measures (eg, mobility) or biomarkers (eg, amyloid-beta and tau).

Thirdly, we also encountered a lot of negative values in our reward. It could be the result of the small dataset, inconsistent data entry for MMSE scores for patients, and the high number of missing values in the record. We tried to minimize the missing values by filling the missing spot with the data from previous visits. The rewards would be better if accurate MMSE scores were present for each visit for all the patients.

Lastly, there was no active RL environment to test our algorithms as it is almost impossible to have an active testing environment for medical patients. Off-policy RL algorithms are only successful when they receive direct feedback from an active environment (eg, a video game). In addition, we do not have sufficient data to perform a thorough confounding factor analysis with respect to factors such as age, medical history, etc. With a proper dataset with evenly distributed medications and fewer missing values, we could use highly effective offline RL algorithms like CQL in the future to avoid this problem.^39^

## CONCLUSIONS

While there are a plethora of studies using AI techniques for the diagnosis of AD, there is a lack of methods applied for learning treatment regimens. In this article, we presented 2 RL techniques for learning treatment regimens for AD. In particular, the policy iteration and Q-Learning methods were used to learn the treatment regimens. We used 2 large open-source longitudinal databases—ADNI and AIBL for this purpose. The ADNI dataset was used for training, validation, and testing. The AIBL dataset was used for testing the generalizability of the models. Our results demonstrate that RL has the potential to learn treatment policies whose outcome is comparable to or better than clinician policies.

## Supplementary Material

ocad135_Supplementary_Data

## Data Availability

The data can be accessed via https://adni.loni.usc.edu/data-samples/access-data/. The used code is publicly available at https://github.com/bioIKEA/Treatment_optimization_AD.

## DATA PERMISSIONS

We have received permission from the Laboratory of Neuro Imaging (LONI) for the utilization of the ADNI and AIBL databases for our nonclinical and IRB-exempt research.
