# Supplementary material for: Using artificial intelligence to learn optimal regimen plan for Alzheimer’s disease
Source: J Am Med Inform Assoc. 2023 Jul 18;30(10):1645–56. doi: 10.1093/jamia/ocad135 (PMC10531148; doi:10.1093/jamia/ocad135)
Supplement: ocad135_Supplementary_Data [file ocad135_supplementary_data.docx]

**Using Artificial Intelligence to Learn Optimal Regimen Plan for Alzheimer's Disease**

**Kritib Bhattarai^1,※^,** **Sivaraman Rajaganapathy^4,※^, Trisha Das^2^, Yejin Kim^3^, Yongbin Chen^4^, the Alzheimer’s Disease Neuroimaging Initiative^†^, the Australian Imaging Biomarkers and Lifestyle flagship study of ageing^‡^, Qiying Dai^4^, Xiaoyang Li^4^, Xiaoqian Jiang^3^, Nansu Zong^4, *^**

^1^Luther College, Decorah, Iowa, United States; ^2^University of Illinois Urbana-Champaign, Champaign, Illinois, United States; ^3^University of Texas Health Science Center, Houston, Texas, United States; ^4^Mayo Clinic, Rochester, Minnesota, United States;

^※^ The authors contributed equally;

^*^Corresponding author, Nansu Zong, Ph.D. ([*zong.nansu@mayo.edu*](mailto:zong.nansu@mayo.edu))

***Supplement 1***

**
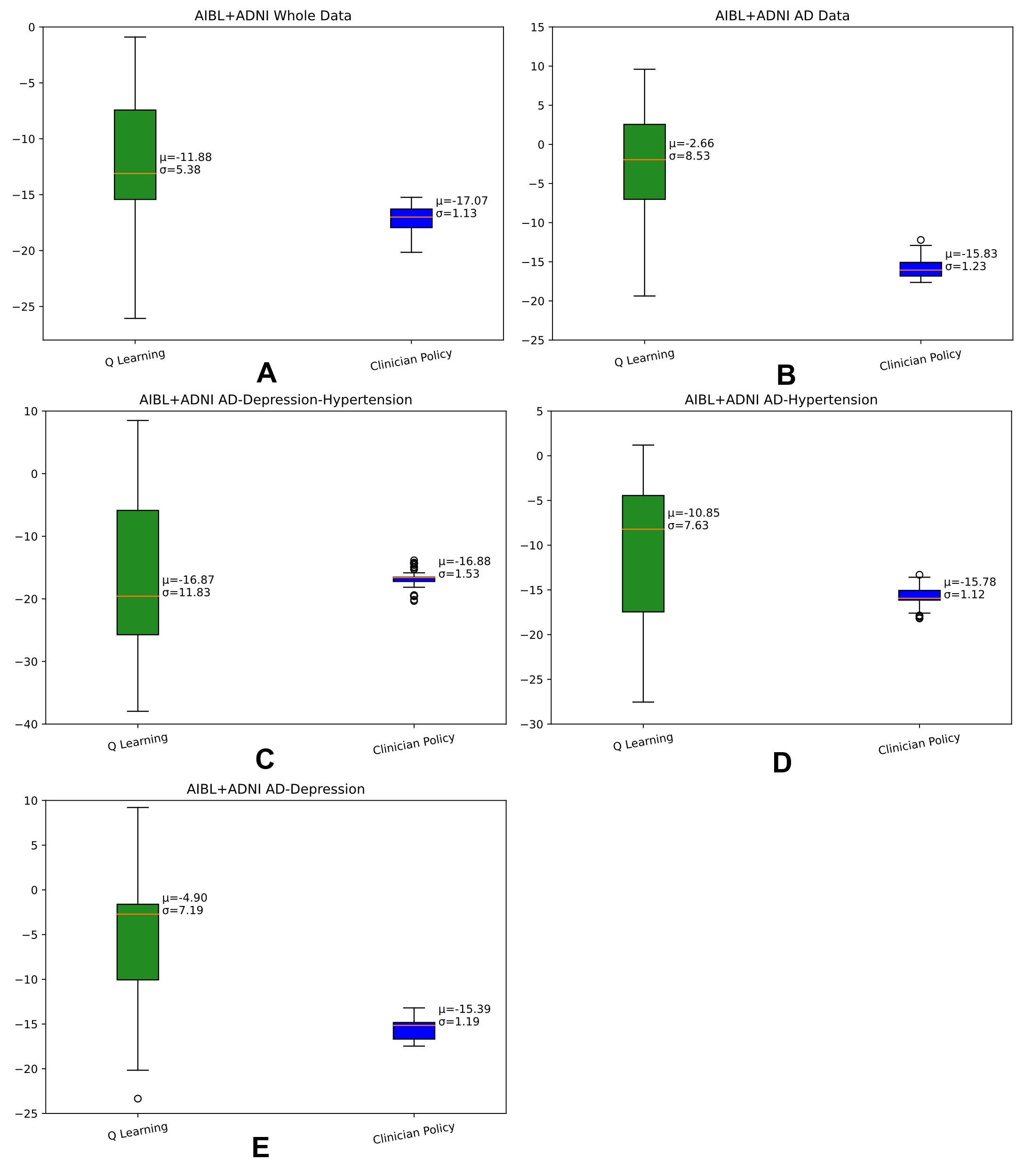
**

**Supplement Figure 1.**  Testing the Q-Learning model on the AIBL test data

***Supplement 2***

*
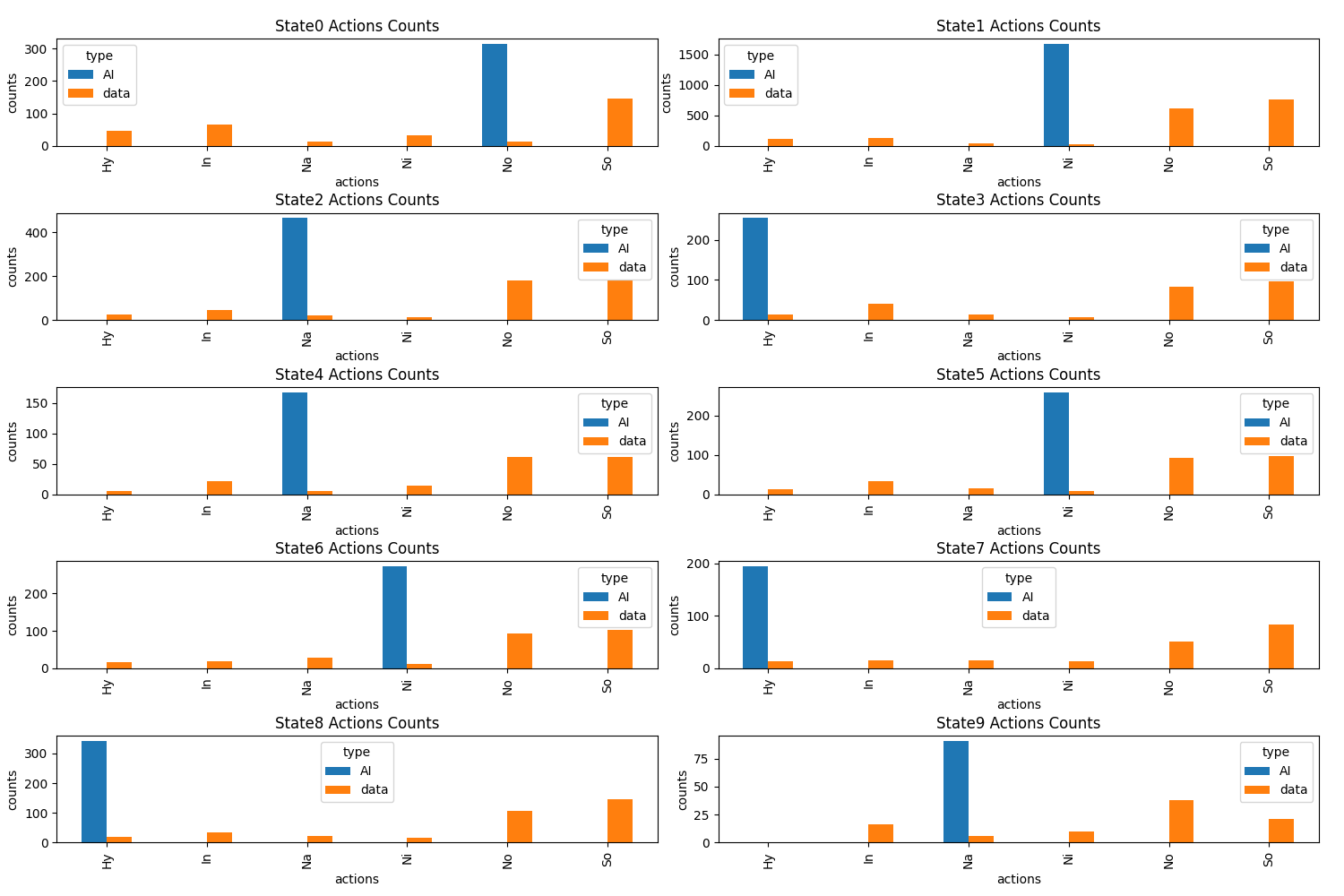
*

**Supplement Figure 2.** Drugs (actions) recommendation counts for each state by Q-learning and Clinician’s policy for AD-Hypertension-Depression Cohort. In X-axis, No is no drugs, In is inhibitors, Me is memantine, Hy is hypertension drugs, Ni is the combination of Memantine and inhibitors and So is supplements/other drugs.

***Supplement 3***


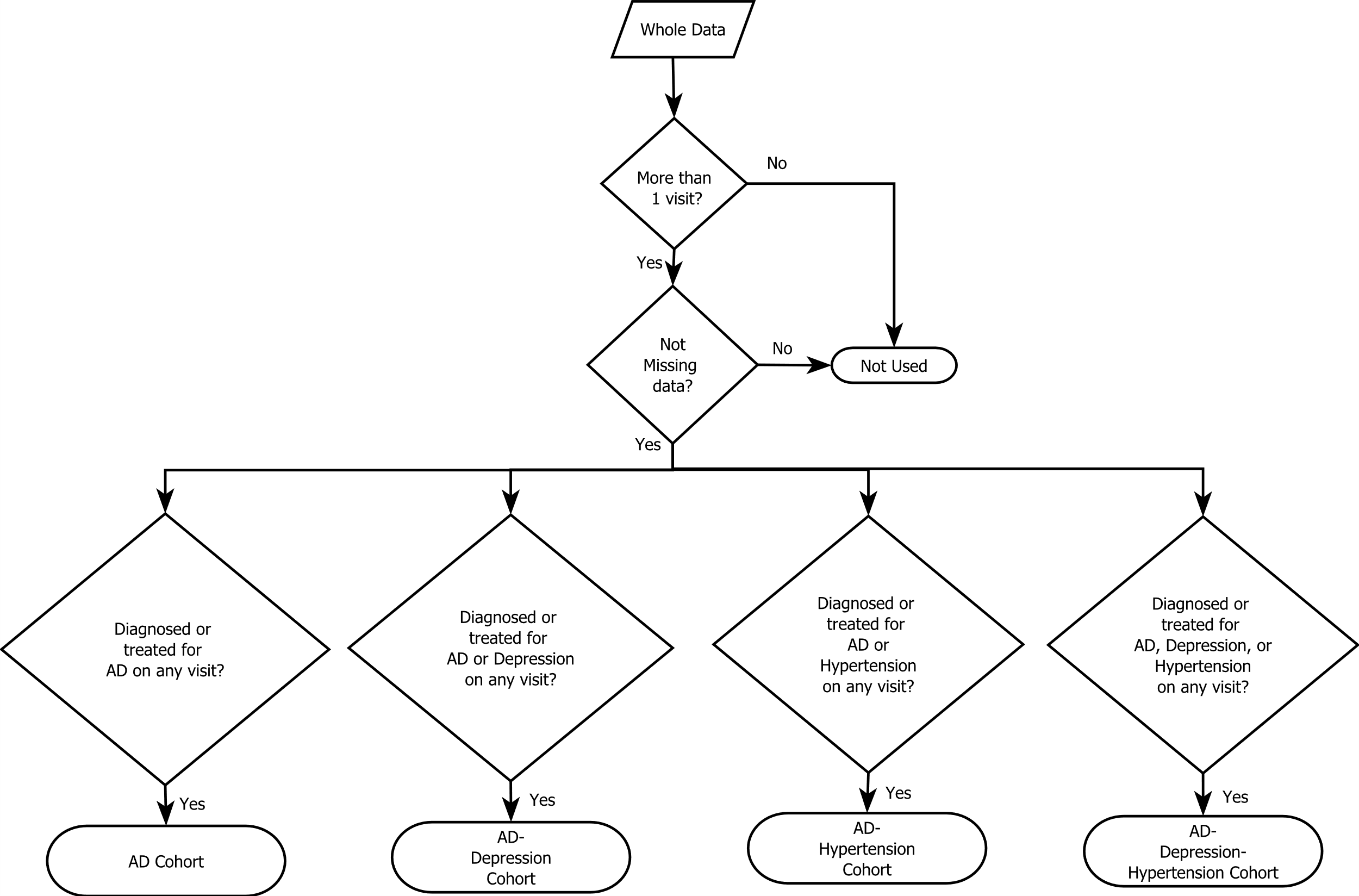


**Supplement Figure 3.** Flow chart showing patient selection criteria

***Supplement 4***

*
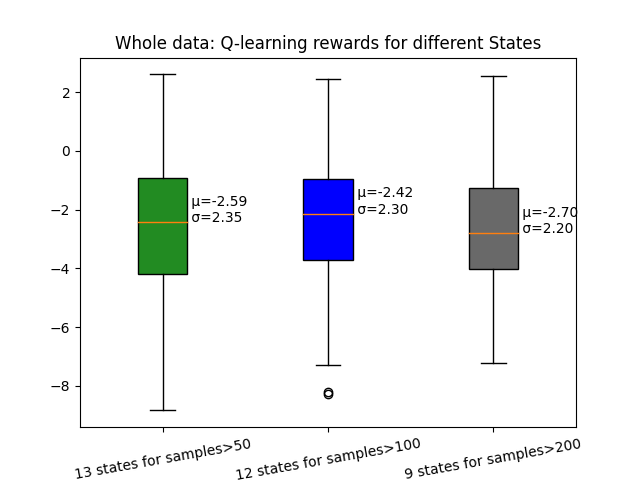
*

**Supplement Figure 4.** Comparison of Q-learning policy for the different number of states for whole data. The number of states is based on the number of samples on the leaf node of a decision tree.

***Supplement 5***

*
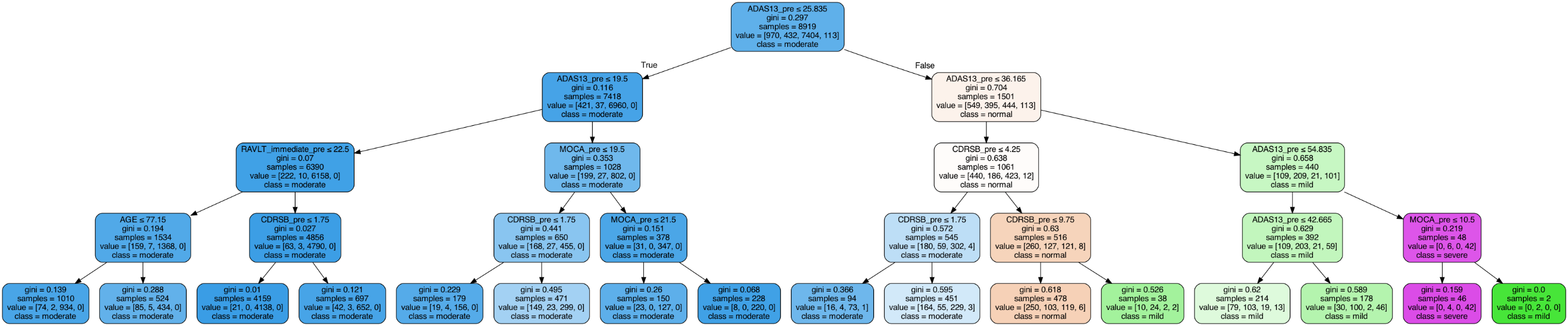
*
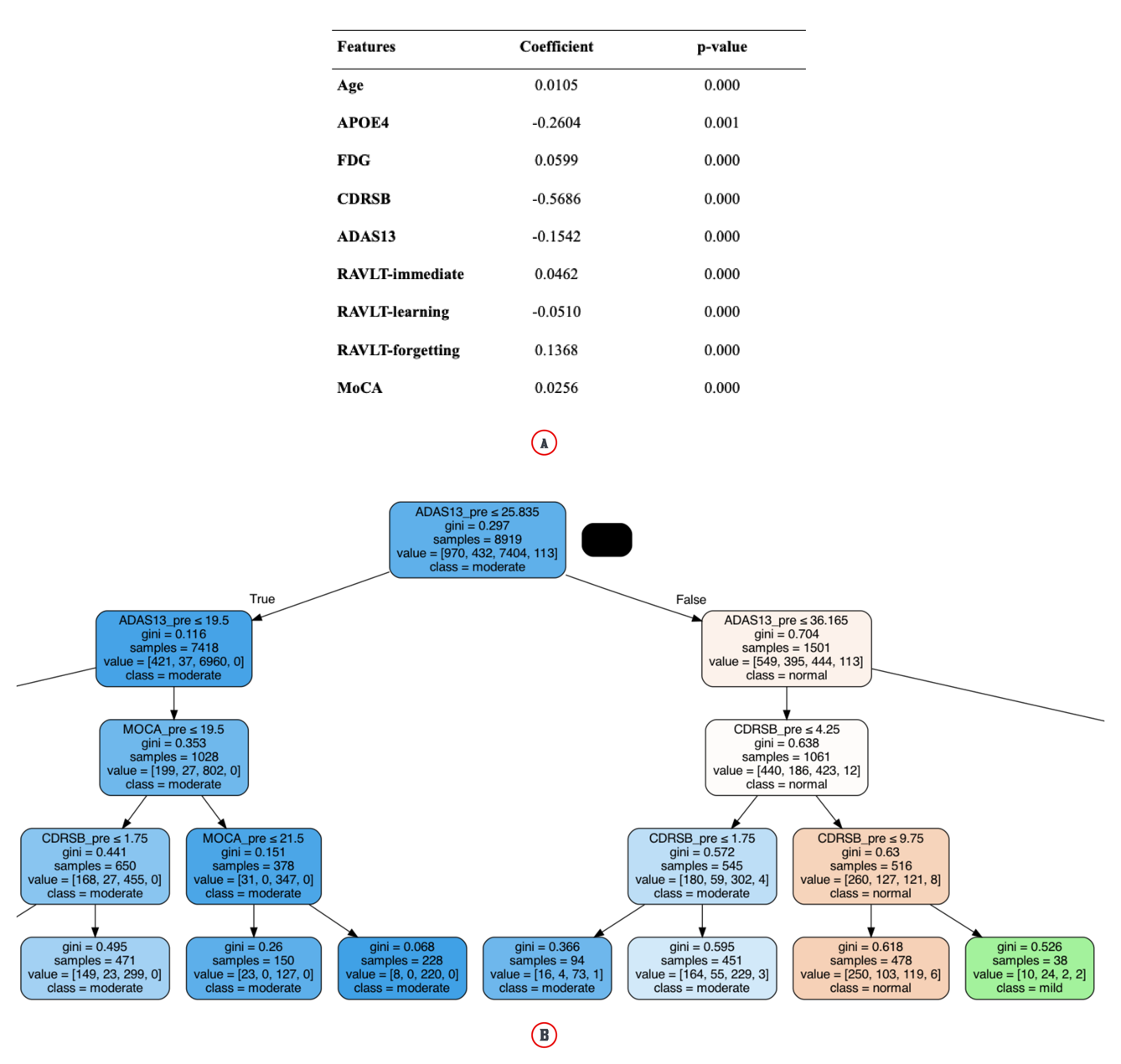


(A)

(B)

**Supplement Figure 5.** (A) Linear Regression table to find the statistically significant features. The significant features are then used for decision tree regression to classify data into different states. (B) Decision tree to predict MMSE scores.

***Supplement 6***


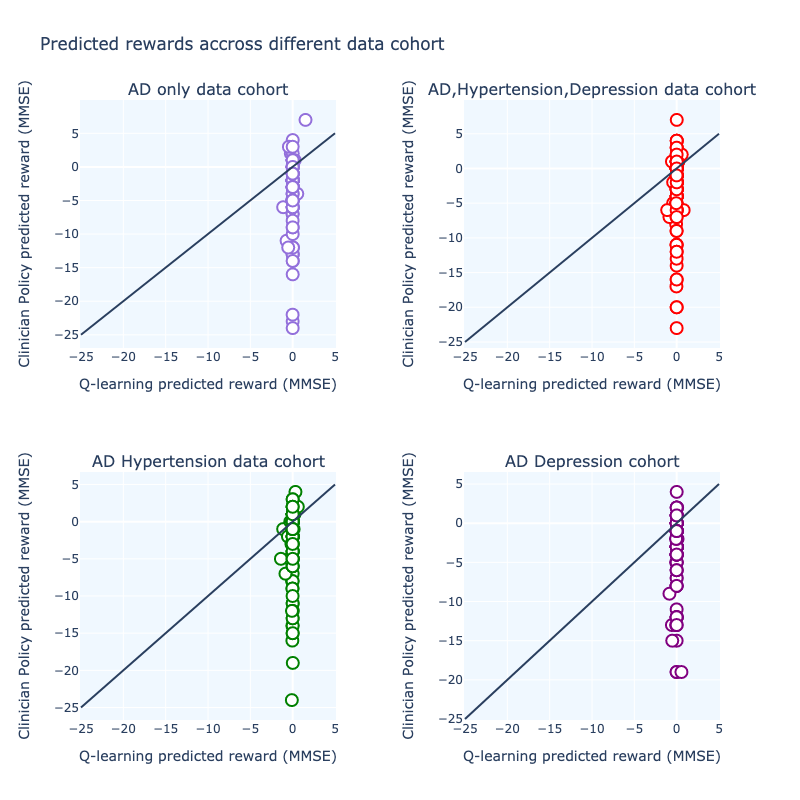


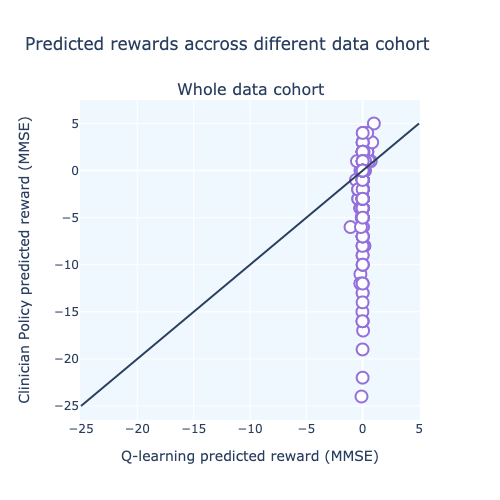


**Supplement Figure 6.** Comparison of reward prediction for different states between Q-learning and clinician’s policy for different data cohorts.

***Supplement 7***


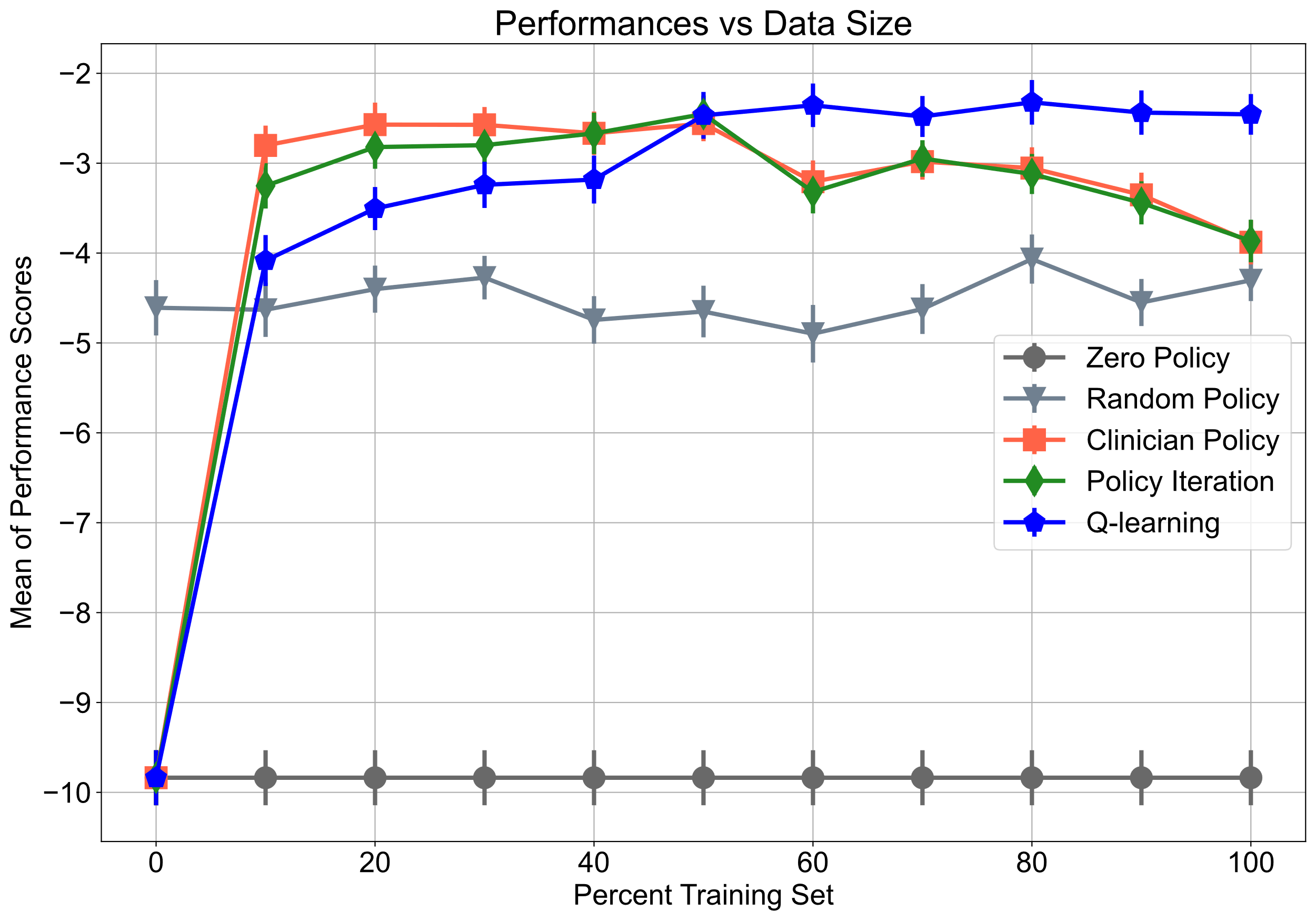
**Supplement Figure 7.** Comparison of the performances of the different policies as the size of the training data is increased. The plot shows the mean of the performance scores, while the error bars show the standard error in the mean. It can be seen that the Clinician Policy, Policy Iteration method, and the Q-learning methods improve in performance as the size of the training data is increased. The Q-learning method has better performance compared to the clinician policy when more than 50% of the training data is used.
